# Generating better leaf traits in M2 lines of fourteen Ethiopian sesame (*Sesamum indicum* L.) genotypes through the treatment of their seeds with sodium azide

**DOI:** 10.1016/j.heliyon.2022.e11984

**Published:** 2022-12-01

**Authors:** Micheale Yifter Weldemichael, Yemane Tsehaye Baryatsion, Desta Berhe Sbhatu, Girmay Gebresamuel Abraha, Hagos Mohammedseid Juhar, Abraha Birhan Kassa, Fiseha Baraki Sibhatu, Hailay Mehari Gebremedhn, Tesfakiros Semere Gebrelibanos, Mohammed Mebrahtu Mossa, Birhanu Debesay Berhe, Haftay Abadi Gebru

**Affiliations:** a Mekelle University, P.O. Box 231, Mekelle, Ethiopia; b Humera Agricultural Research Center, P.O. Box 523, Humera, Ethiopia; c Tigrai Biotechnology Center Pvt. Ltd. Co., P.O. Box 223, Mekelle, Ethiopia

**Keywords:** Clustering, Qualitative leaf traits, Quantitative leaf traits, *Sesame indicum*, Sodium azide

## Abstract

The present study explored the effect of sodium azide (NaN_3_) on quantitative and qualitative leaf traits of M2 lines on 14 Ethiopian sesame genotypes collected from Humera Agricultural Research Center, Tigrai, Ethiopia. Qualitative data on leaf color, leaf hairiness, leaf arrangement, leaf shape, basal leaf profile, basal leaf margin, and leaf angle to main stem as well as quantitative data on length of basal leaf, length of top leaf, width of basal leaf, width of top leaf, length of marginal leaf, and width of marginal leaf were recorded and analyzed using analysis of variance, clustering analysis, Mahalanobis distance, and principal component analysis. Generally, treatment of seeds with NaN_3_ has brought many distinct and statistically significant phenotypic changes on both quantitative and qualitative leaf traits of the M2 lines. The changes in the NaN_3_ treated and locally adapted genotypes of Gumero and Zeri Tesfay are promising; producing the highest mean length of basal leaf (*p* ≤ 0.01). NaN_3_ treated seeds of Baha Necho, Gumero, and Hirhir developed the highest mean width of basal leaf. Locally adapted genotypes have responded positively to NaN_3_ treatment, generating better leaf traits as compared to the research improved ones. This study was the first of its kind in exploring the effects of NaN_3_ seed treatment on leaf traits of sesame genotypes. The findings of this study will, therefore, serve as a steppingstone to look into the effects of the changes in sesame yield and initiate future genetic and molecular studies on the responsive genotypes.

## 1 Introduction

Sesame (*Sesamum indicum* L.) (*Sesamum*, Pedaliaceae) is a major oilseed crop widely cultivated all around the world (Zhou et al., 2018). The production and productivity of this important oilseed crop is very low because of various biotic and abiotic stresses. Yet, research and development programs aiming at improving the production and productivity of the crop are inadequate due to lack of funding, absence of continuous and comprehensive research programs for its improvement, and limited national and international cooperation for its germplasm exchanges. Very limited attention has been given to its genetic improvement and, hence, it is regarded as an orphan crop (Dossa et al., 2017a). Advanced research at genetic and molecular level are restricted to exploring the effects of abiotic and biotic stresses. Abiotic stresses such as drought (Dossa et al. 2016a, 2016b, 2017b, 2017c, 2019a, 2019b, 2020; Wang et al. 2016, 2018b; Li et al., 2017; Mmadi et al., 2017; Zhang et al., 2018; You et al., 2019), waterlogging and salinity (Wei et al., 2019; Zhang et al., 2019) were found to affect the biomass and morphology of its roots and its nutritional composition (Wang et al., 2018a). Many other studies have also shown significant loss of sesame yield due to biotic stresses (Li et al., 2012; Wei et al., 2016; Wang et al., 2017; Asekova et al. 2020, 2021; Yan et al., 2021).

In light of a predicted reduction in agricultural productivity of many crops across the world due to climate change, crop improvement through induced mutation technology has become an increasingly promising area of research over the last few decades (Dubey et al., 2017). Therefore, chemical mutagens have now become useful tools in crop improvement. Studies have shown the potential of mutagens in producing varieties tolerant or resistance to abiotic and biotic stresses in various susceptible crops while improving their yield and quality traits (Olawuyi and Okoli, 2017). To date, several mutagens, including NaN_3_ are being adapted for crop improvement with positive and negative effects (Heslot, 1977). Mutagens help us to understand the mechanism of mutation induction and quantify the frequency and patterns of changes in plants. Mutation breeding helps to build and expand knowledge base and technology towards perfecting better tools for crop improvement. Studies aiming at establishing the efficacies of NaN_3_ in inducing mutagenesis in sesame are, however, limited. The present research, therefore, has looked into the effects of this mutagen in sesame improvement.

An optimal leaf shape is important to avoid self-shading and maintain the ideal plant architecture for high yield (Stewart et al., 2003). Moreover, crop yield is affected by several traits such as number of seeds per capsule, number of capsules per plant, seed weight, capsule dimensions, plant height, height of the first capsule axis, number of internodes, branching type, capsule shattering, and plant growth habit as well as management practices and biotic and abiotic factors (Student, 2008; Diouf et al., 2010; Wu et al., 2014). Scientists pursue research to identify high yielding genotypes with quality traits among cultivars, introgressed lines, and wild relatives. Thus, photoperiod sensitivity becomes one of the primary targets of improvement in crop breeding. Leaf related traits of sesame are very essential in affecting photosynthesis that leads to changes in yield and yield related traits. However, basic and applied research programs for sesame improvement through mutation breeding using NaN_3_ towards improving leaf traits are very limited compared to other crops. Therefore, this research aims to examine the effect of NaN_3_ on the quantitative and qualitative leaf traits of M2 lines on 14 Ethiopian sesame (*S. indicum*) genotypes.

## 2 Materials and methods

### 2.1 Experimental site

The study was carried out at Tigrai Biotechnology Center, Pvt. Ltd. Co. in Mekelle City, Tigrai, northern Ethiopia (Lat.: 13°30′0″N; Long.: 39°28′11″E; Alt. 2,080 masl).

### 2.2 Collection of seeds and sterilization

Seed samples of 14 sesame genotypes were obtained from Humera Agricultural Research Center, Tigrai containing an introduced variety from Israel (i.e., ADI), eight released varieties from different research centers in Ethiopia (i.e., Setit 1, Setit 2, Humera 1, Gondar 1, Borkena, Baha Zeyit, Baha Necho and ACC44), and five local collections acquired from the Ethiopian Biodiversity Institute (i.e., Zeri Tesfay, Hirhir, Gumero, Bounji and Aberghele). The seeds were disease-free, normal shaped, dry and quiescent. After washing with tap water for 20 min, all seeds were soaked in a detergent solution called teepol with 3% for 5 min. Finally, the seeds were rinsed with distilled water. This was followed by disinfecting the seeds with 70% ethanol for 45 s and finally rinsed three times with sterile distilled water. Then, concentrated H_2_SO_4_ was used to treat the seeds for 15–20 min on Petri dishes and were sterilized with 10 mL glacial acetic acid mixed with 20 mL commercial bleach in a closed desiccator for 60 min (Smith et al., 2002). Finally, the seeds were sterilized by soaking them in sterile distilled water for 16 h.

### 2.3 Treatment with sodium azide

Seeds were treated with Sörenson phosphate solution (Sigma-Aldrich, Munich, Germany) containing 0.75% NaN_3_ (Kiran Light Laboratories, Mombai-400002, India) at pH 3 after pre-soaking in cold-tap water at 4 °C for 24 h (Tripathy et al., 2019). After rinsing the seeds with sterile distilled water repeatedly, each genotype was soaked in 10 mL of sterile distilled water overnight. The following day, the seeds were air dried and washed in running tap water for 4 h to remove excess chemical residues. Besides, untreated seeds were included as control. Then, both treated and untreated (control) seeds were sown in greenhouse beds to generate M1 seeds. Finally, M2 plants were generated from clean, good-looking and healthy seeds obtained from the M1 plants.

### 2.4 Evaluation of M2 lines

Of the 14 genotypes, M2 plants with better germination and vegetative growth under greenhouse conditions that demonstrated desirable traits were selected based on quantitative and qualitative data information. M2 seeds of the selected genotypes were planted on 2 m × 2 m plots in the greenhouse with inter and intra-raw spacings of 40 cm and 10 cm, respectively. Planting was carried out using a completely randomized design (CRD) replicated thrice. All other necessary agronomic practices were carried out during the growth period of the plants as necessary. Quantitative and qualitative data were collected at 75% maturity. Recorded quantitative data included: length of basal leaf (LBL), length of top leaf (LTL), width of basal leaf (WBL), width of top leaf (WTL), length of marginal leaf (LML), and width of marginal leaf (WML). Likewise, recorded qualitative data were: leaf color (LC), leaf hairiness (LH), leaf arrangement (LA), leaf shape (LS), basal leaf profile (BLP), basal leaf margin (BLM), andleaf angle to main stem (LAMS) (Table 1).

**Table 1:** Descriptors recorded for qualitative traits of M2 lines of *S*. *indicum*.

SN	Descriptor	Scoring
1	Leaf color	1: Green; 2: Green with yellowish cast; 3: Green with blue-grey cast; 4: Green with purple cast; 5: Other
2	Leaf hairiness	1: Glabrous; 2: Weak or sparse; 3: Medium; 4: Strong or profuse
3	Leaf arrangement	1: Opposite; 2: Alternate; 3: Ternate; 4: Mixed
4	Leaf shape	1: Linear; 2: Lanceolate; 3: Elliptic; 4: Ovate; 5: Narrowly cordate; 6: Other
5	Basal leaf profile	1: Flat; 2: Cup shaped (concave); 3: Reverse cup shaped (convex)
6	Basal leaf margin	1: Entire; 2: Serrate; 3: Dentate
7	Leaf angle to main stem	1: Acute; 2: Horizontal; 3: Dropping

Source: (IPGRI 2004).

### 2.5 Data analyses

The collected data for the traits measured were subjected to analysis of variance (ANOVA) using GenStat 16 Software (Payne et al., 2002). Post-ANOVA mean comparisons were carried out using Duncan's Multiple Range Test at *a priori* set significance level of *p* ≤ 0.01 (Duncan, 1955). The generated data were also used to carry out genotype grouping through clustering analyses, Mahalanobis distance, and principal component analysis.

## 3 Results and discussion

Leaves are the most important organs of majority of the vascular plants, which capture solar energy from sun light to synthesize carbohydrate from water and carbon dioxide through photosynthesis. Altering the size of the leaves will hence bring an effect on their capacity to photosynthesize and yield. This study showed that NaN_3_ treatment has significantly affected all the leaf traits measured including length of basal leaf (LBL), length of top leaf (LTL), width of top leaf (WTL), length of marginal leaf (LML), width of basal leaf (WBL), and width of marginal leaf (WML).

### 3.1 Effects of NaN_3_ on quantitative leaf traits of M2 lines

The ANOVA clearly indicated that treatment of seeds with NaN_3_ showed highly significant differences among the genotypes of the M2 lines for all of the leaf traits measured, except for LTL. The genotypes, concentrations of NaN_3_ and their interaction effect showed highly significant effects on the quantitative leaf traits such as length of basal leaf (LBL), length of top leaf (LTL), and width of top leaf (WTL) (*p* ≤ 0.01; Table 2). On the other hand, the remaining quantitative leaf traits including width of basal leaf (WBL), length of marginal leaf (LML), and width of marginal leaf (WML) had no interaction effect (Table 2).

**Table 2:** ANOVA results on leaf traits of M2 *S. indicum* plants.

Source of variation	*df*	LBL	LTL	WBL	WTL	LML	WML
Variety	13	45.633∗∗	1.663ns	12.379∗∗	1.2701∗∗	42.122∗∗	15.50∗∗
Treatment	1	138.857∗∗	33.063∗∗	61.543∗∗	16.8305∗∗	319.379∗∗	53.037∗∗
Variety × Treatment	13	8.513∗∗	4.930∗∗	2.683^ns^	1.7891∗∗	6.649^ns^	2.539^ns^
Residual	84	3.189	1.212	1.711	0.3699	7.491	2.319

LBL: length of basal leaf; LTL: length of top leaf; WBL: Width of basal leaf; WTL: width of top leaf; LML: length of marginal leaf; WML: width of marginal leaf. ∗∗: *p* ≤ 0.01; ∗: *p* ≤ 0.05; ns: non-significant.

#### 3.1.1 Length of basal leaf

The highest mean LBL was observed on the locally adapted, NaN_3_ treated genotypes; namely, Gumero and Zeri Tesfay. On the other hand, the lowest mean LBL was observed on the treated (Setit 1, Setit 2, Borkena, and ACC44) as well as the control (Setit 2 and Setit1) genotypes (Table 3). All these genotypes are research improved, released from different research centers in Ethiopia. The present study also revealed that the locally adapted genotypes have higher mean LBL values compared to the released ones. Mutant traits are always useful in genetics and breeding of sesame. Such traits demonstrate distinct features in sesame phenotypes such as leaves (e.g., narrow, elongated, or thick), petioles (e.g., short or elongated), and flowers (e.g., white or pigmented).

**Table 3:** Interaction effect of NaN_3_ treatment on different quantitative leaf traits of M2 *S. indicum* lines.

Genotypes	LBL		LTL		WBL		WTL		LML		WML	
	Treated	Control	Treated	Control	Treated	Control	Treated	Control	Control	Treated	Control	Treated
Aberghele	12.00^c-f^	12.33^b-f^	3.933^b-d^	7.778^a^	7.363 ^a-e^	5.728 ^a-e^	1.804^e^	3.694^a-d^	11.67^e-k^	15.40^b-f^	6.00^c-j^	8.60^a-c^
ACC44	11.10^c-f^	8.50^ef^	5.173^a-d^	4.144^b-d^	6.363 ^a-e^	4.894^c-e^	2.704^b-e^	2.494^b-e^	10.00^g-k^	16.00^d-e^	5.00^e-j^	7.10^b-h^
ADI	10.70^c-f^	10.67^c-f^	3.773^cd^	4.878^a-d^	5.963 ^a-e^	6.228 ^a-e^	1.744^e^	2.694^b-e^	12.00^e-k^	11.90^e-k^	4.83^f-j^	5.30^e-j^
Baha Necho	14.00^a-d^	10.83^c-f^	4.733^a-d^	6.478^a-d^	8.863^ab^	6.394 ^a-e^	2.364^c-e^	2.927^b-e^	12.67^c-k^	16.90^a-d^	7.33^a-f^	9.70^a^
Baha Zeyit	12.60^c-e^	10.67^c-f^	4.153^b-d^	5.744^a-d^	7.463^a-e^	6.228 ^a-e^	2.044^de^	4.127^a-c^	11.83^e-k^	15.40^b-f^	6.00^c-j^	8.20^a-c^
Borkena	11.60^c-f^	8.00^ef^	4.613^a-d^	5.211^a-d^	6.863 ^a-e^	4.094^de^	1.904^de^	2.127^de^	10.33^g-k^	14.20^c-j^	5.50^d-j^	7.60^a-e^
Bounji	15.20^a-c^	12.83^a-d^	4.793^a-d^	6.711^a-c^	7.463 ^a-e^	6.561 ^a-e^	2.704^b-e^	2.860^b-e^	14.33^b-i^	16.80^a-d^	6.83^b-i^	7.30^a-f^
Gondar 1	14.00^a-d^	11.50^c-f^	4.353^b-d^	6.111^a-d^	8.363^a-c^	6.561 ^a-e^	2.124^de^	3.194^a-e^	11.17^e-k^	15.00^b-f^	6.33^b-j^	7.60^a-e^
Gumero	18.10^a^	9.67^d-f^	4.013^b-d^	6.944^a-c^	9.263^a^	5.561 ^a-e^	2.384^c-e^	3.127^a-e^	12.17^d-k^	18.60^ab^	6.17^b-j^	8.50^a-c^
Hirhir	11.90^c-f^	9.17^def^	5.733^a-d^	4.611^a-d^	9.063^ab^	5.228^b-e^	2.184^de^	4.927^a^	10.83^f-k^	14.20^c-j^	6.00^c-j^	5.30^e-j^
Humera 1	14.40^a-d^	11.67^c-f^	4.853^a-d^	6.278^a-d^	8.063^a-c^	6.894 ^a-e^	2.304^de^	4.360^ab^	12.33^c-k^	17.00^a-c^	6.00^c-j^	8.70^ab^
Setit 1	8.30^ef^	7.67^ef^	4.173^b-d^	4.011^b-d^	4.263^de^	4.061^de^	1.704^e^	2.327^c-e^	10.00^g-k^	10.70^g-k^	3.83^j^	4.30^ij^
Setit 2	8.20^ef^	6.83^f^	4.713^a-d^	3.944^b-d^	4.563^de^	3.728^e^	2.524^b-e^	1.860^de^	9.00^jk^	10.20^i-k^	4.50^f-j^	4.00^j^
Zeri Tesfay	17.20^ab^	13.00^a-d^	3.373^d^	7.111^ab^	7.763^a-d^	5.561 ^a-e^	1.924^de^	2.227^de^	15.33^b-f^	20.20^a^	6.07^c-j^	8.10^a-d^
CV	15.1		22.3		19.8		24.3		19.7		23	

LBL: length of basal leaf; LTL: length of top leaf; WBL: width of basal leaf; WTL: width of top leaf; LML: length of marginal leaf; WML: width of marginal leaf. Mean values in the same column with different letters are significantly different at *p* ≤ 0.01.

#### 3.1.2 Length of top leaf

The analysis of variance revealed that treatment of the seeds with NaN_3_ has significantly affected the LTL of the genotypes (*p* ≤ 0.01). It significantly increased the mean LTL in ACC44, Hirhir, Setit 1, and Setit 2, but reduced in the remaining 10 genotypes. The highest mean LTL values were observed on the genotypes used as control, with the top two being Aberghele (7.778 cm) and Zeri Tesfay (7.111 cm) both locally adopted genotypes. NaN_3_ treatment also revealed the lowest mean LTL in ADI (research improved) and Zeri Tesfay (locally adapted) genotypes.

#### 3.1.3 Width of basal leaf

NaN_3_ treatment significantly increased the mean WBL in all of the tested genotypes except in ADI. The highest mean WBL values were observed on the treated genotypes of Gumero (9.263 cm), Hirhir (9.063 cm), and Baha Necho (8.863 cm). On the other hand, the lowest mean WBL values were recorded in the untreated (control), research improved genotypes of Setit 2, Setit 1, and Borkena, while the untreated (control) ADI genotype produced the highest WBL.

#### 3.1.4 Width of top leaf

The highest mean WTL values were recorded in the untreated seeds of Hirhir and Humera 1 genotypes. On the other hand, the lowest mean WTL values were observed on the treated genotypes of Aberghele, ADI, Baha Zeyit, Borkena, Gondar 1, Hirhir, Humera 1, Setit 1, and Zeri Tesfay as well as on the untreated (control) genotypes of Borkena, Setit 2, and Zeri Tesfay.

#### 3.1.5 Length of marginal leaf

The highest mean LML values were recorded in the treated genotypes of Zeri Tesfay and Gumero. On the other hand, the lowest mean LML value was observed on the untreated seeds of Setit 2 genotype.

#### 3.1.6 Width of marginal leaf

The highest mean WML values were recorded in the treated genotypes of Baha Necho and Humera 1, while the untreated seeds of Setit 1 genotype yielded the lowest mean value of WML.

In summary, this study revealed that treatment of sesame seeds with NaN_3_ led to desirable changes in multiple traits of leaf morphology, which would enhance the light harvesting capacity and photosynthetic efficiency of the crop towards yield improvements. Mutations that positively changed M2 sesame leaf morphology towards desirable agronomic traits were induced by ethyl methanesulphonate (EMS) elsewhere (Jayabalan 1995). Lines or mutants with thick-leaves are most preferred as they possess superior agronomic traits including more branches per plant, more capsules on the main axis, longer distance from base to first branching, more capsules per plant, higher seed yield, and higher seed protein content as compared to the parent variety (Najeeb et al., 2012).

**Figure 1:**
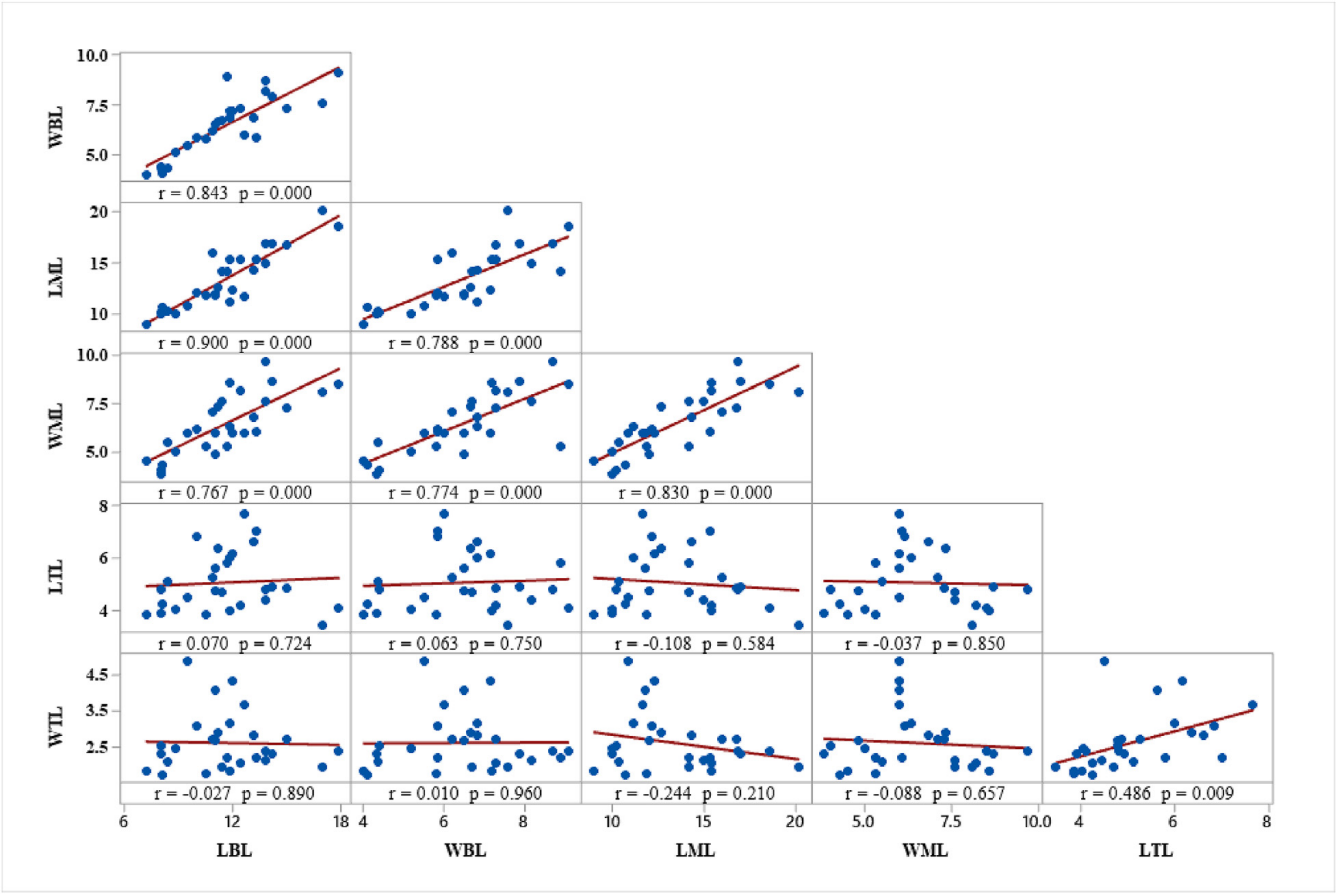
Matrix plot for the quantitative leaf traits of sesame genotypes.

Correlation analysis is useful to understand the association among different phenotypic traits allows plant breeders to select genotypes having desirable traits that can be selected directly or indirectly for crop improvement programs. In this study, phenotypic correlation analysis was done for the six quantitative traits of sesame (Figure 1). Most of the correlation coefficients showed positive and highly significant (P < 0.001) values that ranged from *r* = 0.77 to r = 0.90. The strong positive correlations were observed between LBL and LML, LBL and WBL, LBL and WML, LML and WBL, LML and WML, and WBL and WML. Those highly correlated phenotypic traits should be considered and can easily be selected together during selection for leaf trait improvements of sesame. Moderate and significant correlation coefficient (*r* = 0.49, P > 0.01) was found between the top leaf traits, LTL and WTL, but neither of them has significant correlation with the other leaf traits. There was very weak and insignificant correlation among the pairs of the rest traits.

### 3.2 Effects of NaN_3_ on qualitative leaf traits of M2 lines

In this study, the treatment of seeds with NaN_3_ was found to be instrumental to bring many changes in multiple leaf traits of the M2 lines of the tested Ethiopian sesame genotypes. The genotypes, NaN_3_concentrations, and their interaction effect showed significant changes on only four of the seven qualitative leaf traits, namely LS, BLF, BLM and LAMS (Table 4). The findings are interpreted as follows:

**Table 4:** Effect of NaN_3_Treatment of seeds on different qualitative leaf traits of M2 *S. indicum* genotypes (%).

Leaf morphology	Treatment	Name of Genotypes													
		Aberghele	ACC44	ADI	Baha Necho	Baha Zeyit	Borkena	Bounji	Gondar 1	Gumero	Hirhir	Humera 1	Setit 1	Setit 2	Zeri Tesfay
Leaf color															
Green	Control	37.5	37.5	37.5	37.5	37.5	37.5	37.5	37.5	37.5	37.5	37.5	37.5	37.5	37.5
	Treated	62.5	62.5	62.5	62.5	62.5	62.5	62.5	62.5	62.5	62.5	62.5	62.5	62.5	62.5
Leaf hairiness															
Glabrous	Control	37.5	37.5	37.5	37.5	37.5	37.5	37.5	37.5	37.5	37.5	37.5	37.5	37.5	37.5
	Treated	62.5	62.5	62.5	62.5	62.5	62.5	62.5	62.5	62.5	62.5	62.5	62.5	62.5	62.5
Leaf arrangement															
Opposite	Control	37.5	37.5	37.5	37.5	37.5	37.5	37.5	37.5	37.5	37.5	37.5	37.5	37.5	37.5
	Treated	62.5	62.5	62.5	62.5	62.5	62.5	62.5	62.5	62.5	62.5	62.5	62.5	62.5	62.5
Leaf shape															
Lanceolate	Control	100	−	37.5	−	−	37.5	37.5	−	−	−	−	−	37.5	37.5
	Treated	−		62.5			62.5	62.5	−	−	100	−	100.0	62.5	62.5
Ovate	Control	−	37.5	−	37.5	37.5	−	−	37.5	37.5	100	37.5	100.0	−	−
	Treated	100	62.5	−	62.5	62.5	−	−	62.5	62.5	−	62.5	−	−	−
Basal leaf profile															
Flat	Control	−	−	100	37.5	37.5	37.5	−	37.5	37.5	100	37.5	100.0	100	37.5
	Treated	−	100	−	62.5	62.5	62.5	100	62.5	62.5	−	62.5	−	−	62.5
Convex	Control	37.5	100	−	−	−	−	100	−	−	−	−	−	−	−
	Treated	62.5	−	100	−	−	−	−	−	−	100	−	100.0	100	−
Basal leaf margin															
Entire	Control	37.5	100	37.5	−	−	−	37.5	−	−	37.5	−	37.5	37.5	−
	Treated	62.5	−	62.5	100	100	100	62.5	−	−	62.5	−	62.5	62.5	−
Serrate	Control	−	−	−	100	100	100	−	37.5	37.5	−	37.5	−	−	37.5
	Treated	−	100	−	−	−	−	−	62.5	62.5	−	62.5	−	−	62.5
Leaf angle to main stem															
Acute	Control	100	−	−	−	−	−	37.5	100	−	37.5	−	−	100	−
	Treated	−	100	−	−	−	−	62.5	−	−	62.5	100	−	−	−
Horizontal	Control	−	−	−	37.5	37.5	−	−	−	37.5	−	100	−	−	37.5
	Treated	−	−	−	62.5	62.5	100	−	100	62.5	−	−	−	−	62.5
Dropping	Control	−	100	37.5	−	−	100	−	−	−	−	−	37.5	−	−
	Treated	100	−	62.5	−	−	−	−	−	−	−	−	62.5	100	−

#### 3.2.1 Leaf shape

The treatment of seeds with NaN_3_ caused changes in the shapes of the leaves in three genotypes, namely Hirhir, Setit 1, and Aberghele. It changed the leaf shapes of Hirhir and Setit 1 from ovate to lanceolate and that of Aberghele from lanceolate to ovate.

#### 3.2.2 Basal leaf profile

The qualitative data analysis indicated that treatment of the seeds with NaN_3_ brought phenotypic changes in the basal leaf profiles of six genotypes. The flat basal leaf profiles in ADI, Hirhir, Setit 1, and Setit 2 were changed to convex, while the reverse was observed in ACC44 and Bounji.

#### 3.2.3 Basal leaf margin

The treatment of seeds with NaN_3_ caused changes in the basal leaf margin of only four genotypes (Baha Necho, Baha Zeyit, Borkena, and ACC44). It changed the serrate basal leaf margin in Baha Necho, Baha Zeyit and Borkena into entire and the entire in ACC44 into serrate.

#### 3.2.4 Leaf angle to main stem

Likewise, the NaN_3_ treatment brought phenotypic changes of leaf angle to main stem in six genotypes. The changes were from dropping to acute in ACC44, from acute to dropping in Aberghele and Setit 2, from acute to horizontal in Gondar 1, from horizontal to acute in Humera 1, and from dropping to horizontal in Borkena genotypes.

#### 3.2.5 Other qualitative leaf traits

The analysis of variance indicated no impact of NaN_3_ treatment on the other leaf traits such as LC, LH, and LA. Because, the genotypes did not show any change in those leaf characters as the result of NaN_3_ supplement.

### 3.3 Grouping of the genotypes and calculating their Mahalanobis distance

**Figure 2:**
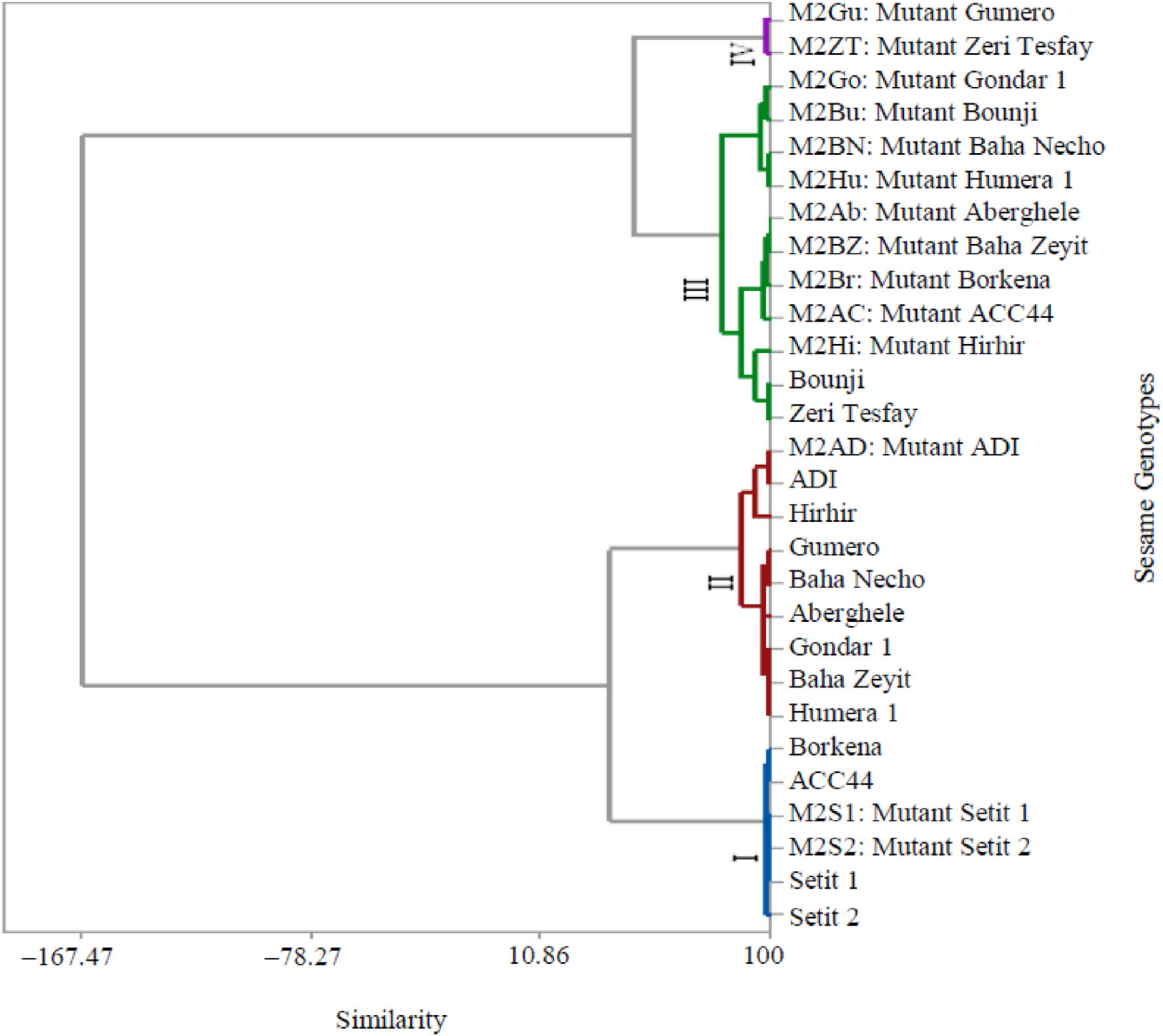
Dendrogram of the combination of untreated and treated genotypes using Ward's method based on dissimilarity matrix.

Comparions of the treated and untraeted genotypes were grouped in this study. The exercise to cluster the M2 lines of the untreated (control) and their corresponding mutant genotypes yielded four distinct clusters (Figure 2). Cluster I included four untreated (i.e., Setit 1, Setit 2, Borkena, and ACC4) and two treated (i.e., Setit 1 and Setit 2) genotypes, which demonstrated the lowest mean values in all the quantitative leaf traits explored in this study. Interestingly, Cluster II included seven untreated (i.e., Humera 1, Baha Zeyit, Gondar 1, Aberghele, Baha Necho, Gumero, Hirhir) and one (ADI) treated genotypes. The mean values of all the quantitative leaf traits of the genotypes of this cluster were relatively moderate. Cluster III comprised 11 genotypes of which two (i.e., Zeri Tesfay and Bounji) were untreated and nine (i.e., Hirhir, ACC44, Borkena, Baha Zeyit, Aberghele, Humera 1, Baha Necho, Bounji, and Gondar 1) were treated. Like in Cluster II, the genotypes in this cluster demonstrated moderate mean values in all the quantitative leaf traits. Finally, Cluster IV included Zeri Tesfay and Gumero, characterized with the highest mean values in all the quantitative leaf traits (Table 5). The genotype grouping generated from the cluster analysis was corroborated by the Mahalanobis distance analysis of clusters (Table 6). The distance values ranged from 16.18 (between I and II) to 23.13 (between II and III) where all the differences were statistically significant (*p* ≤ 0.01).

**Table 5:** Mean quantitative leaf traits of the clusters of the combination of untreated and treated M2 lines.

Clusters	LBL	WBL	LML	WML	LTL	WTL
1	8.072	4.394	10.039	4.522	4.314	2.160
2	11.074	6.311	11.841	5.996	5.753	3.399
3	12.864	7.370	15.506	7.545	5.140	2.301
4	17.450	8.350	19.400	8.300	3.760	2.170

**Table 6:** Mahalanobis distance between clusters of the combination untreated and treated genotypes.

Clusters	1	2	3	4
1	−			
2	16.18∗∗	−		
3	56.22∗∗	23.13∗∗	−	
4	159.61∗∗	102.70∗∗	38.14∗∗	−

*∗∗p* ≤ 0.01.

The variation in the results of clustering based on untreated (3 clusters) and treated (4 clusters) genotypes was also observed elsewhere. Clustering of treated M2 lines based on stem traits has yielded four groups (Weldemichael et al., 2021). Setit 1 and Setit 2 were put in the same cluster based on leaf and stem traits. Clustering based on *in vitro* regeneration of Ethiopian sesame genotypes has also yielded four groups (Weldemichael et al., 2020). The crossing of genotypes in Cluster IV that are characterized by high mean values of all leaf traits with genotypes in Cluster III that are characterized by high yield potential as reported in the work of Baraki et al. (41) can be fruitful in producing early reaching genotypes. Crossing between clusters with wider genetic distance has been suggested to be preferred in developing better heterosis for boosting yield and improve yield related traits in crop improvement (42, 43).

### 3.4 Principal component analysis of data on quantitative leaf traits

**Figure 3:**
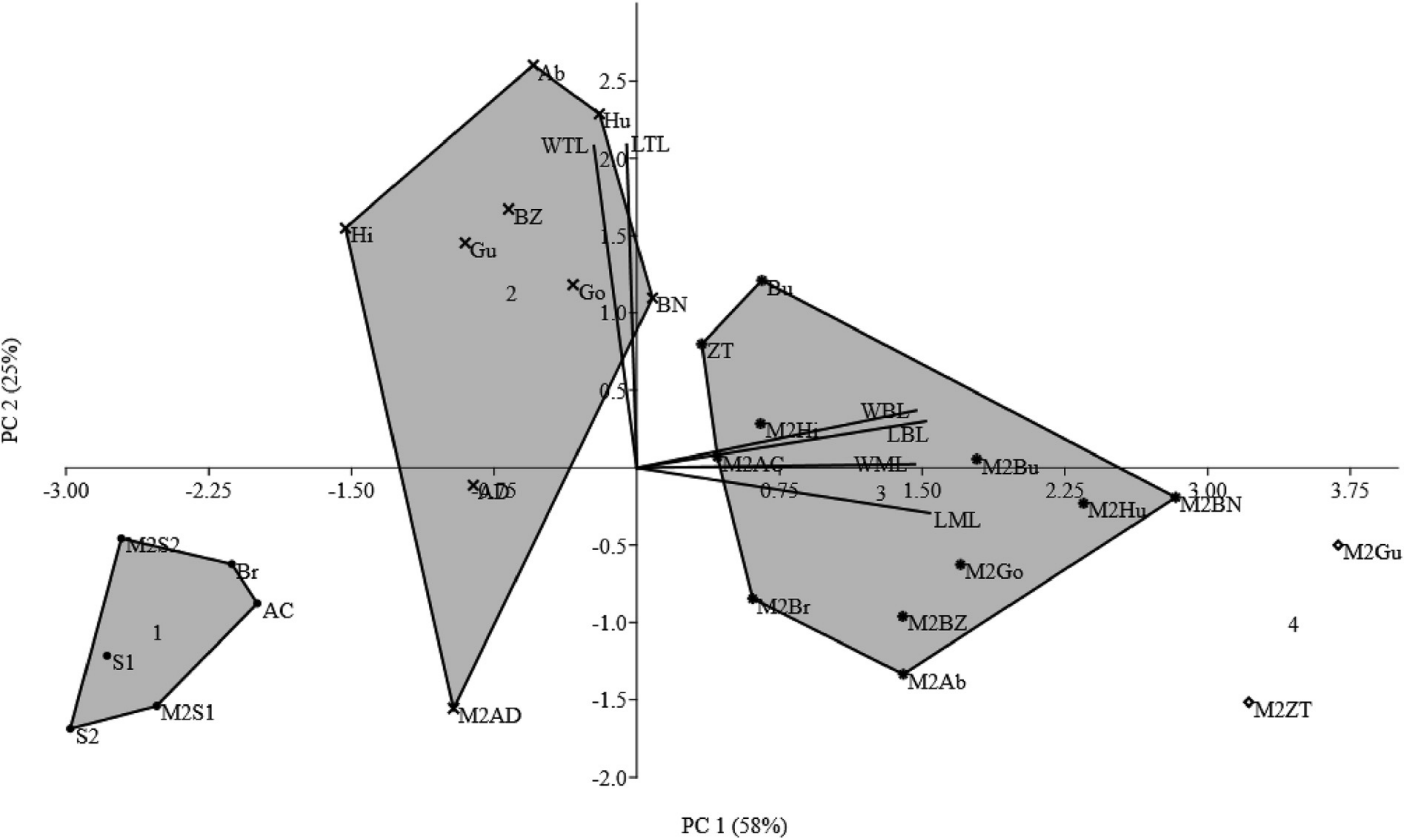
Principal components 1 (X-axis) and 2 (Y-axis) of the variation in the six quantitative leaf traits. Ab: Aberghele; AC: ACC44; AD: ADI; BN: Baha Necho; BZ: Baha Zeyit; Br: Borkena; Bu: Bounji; Go: Gondar 1; Gu: Gumero; Hi: Hirhir; Hu: Humera 1; S1: Setit 1; S2: Setit 2; ZT: Zeri Tesfay; M2Ab: Mutant Aberghele; M2AC: Mutant ACC44; M2AD: Mutant ADI; M2BN: Mutant Baha Necho; M2BZ: Mutant Baha Zeyit; M2Br: Mutant Borkena; M2Bu: Mutant Bounji; M2Go: Mutant Gondar 1; M2Gu: Mutant Gumero; M2Hi: Mutant Hirhir; M2Hu: Mutant Humera 1; M2S1: Mutant Setit 1; M2S2: Mutant Setit 2; M2ZT: Mutant Zeri Tesfay

Principal component analysis (PCA) is one of the oldest and most popular multivariate techniques. The quantitative data of the six leaf traits were subjected to principal component analysis (PCA) (Figure 3). Two out of the six principal components (PCs) had eight values greater than 1, and explained 83% of the total variance. PC 1 (including WBL, WML, LBL, and LML) and PC 2 (WTL and LTL) accounted for 58% and 25% of the variance, respectively. Within the PC 1, the treated Gumero, Baha Necho, Humera 1, Gondar 1, and Bounji genotypes had above average performances in LBL LML, WBL, and WML. One the other hand, the untreated Aberghele and Humera 1 genotypes had high mean values in LTL and WTL, whereas the treated and untreated Setit 1, Setit 2, and ADI genotypes yielded lower mean values in the width and length of basal, marginal, and top leaves within PC 2. The PC has confirmed the correlation between (*a*) WBL and LBL, (*b*) WML and LML, and (*c*) WTL and LTL and absence of significant correlation between the widths and lengths of marginal and top leaves.

## 4 Conclusion

All sesame genotypes, in general, and the Ethiopian sesame genotypes, in particular, are neglected as far as improvement programs using genetic tools are concerned. The present study was aiming at generating genotypes with desirable phenotypic leaf traits to improve the yield of 14 sesame genotypes. In this regard, treatment of sesame seeds with NaN_3_ mutagen produced desirable phenotypic changes in quantitative and qualitative leaf traits of M2 lines. The effects of NaN_3_ treatment were found to be positive and profound in the locally adapted genotypes of Gumero and Zeri Tesfay, indicating their good potential for further research. Treatment of seeds with NaN_3_ is, hence, a promising procedure to generate mutants with better and heritable qualitative and quantitative leaf traits. In the future, research can focus on identification and characterization of mutants based on desired heritable leaf traits using molecular markers. Besides, studies in M3 lines and beyond can help in establishing the distinctness of the mutants that can be generated using this mutagen.

## Declarations

### Author contribution statement

Micheale Yifter Weldemichael: Conceived and designed the experiments; Performed the experiments; Analyzed and interpreted the data; Contributed reagents, materials, analysis tools or data; Wrote the paper.

Yemane Tsehaye Baryatsion, Desta Berhe Sbhatu: Conceived and designed the experiments.

Girmay Gebresamuel Abraha, Hagos Mohammedseid Juhar, Abraha Birhan Kassa, Fiseha Baraki Sibhatu: Performed the experiments.

Hailay Mehari Gebremedhn, Tesfakiros Semere Gebrelibanos, Mohammed Mebrahtu Mossa: Analyzed and interpreted the data.

Birhanu Debesay Berhe, Haftay Abadi Gebru: Contributed reagents, materials, analysis tools or data.

### Funding statement

This work was supported by Mekelle University (No. CRPO/CoDANR/LARGE/001/09).

### Data availability statement

The data that has been used is confidential.

### Declaration of interest's statement

The authors declare no conflict of interest.

### Additional information

No additional information is available for this paper.

## References

[bib1] Asekova S., Oh E., Kulkarni K.P., Siddique M.I., Lee M.H., Kim J.I., Lee J.-D., Kim M., Oh K.-W., Ha T.-J. (2021). An integrated approach of QTL mapping and genome-wide association analysis identifies candidate genes for phytophthora blight resistance in sesame (Sesamum indicum L.). Front. Plant Sci..

[bib2] Asekova S., Oh E., Kulkarni K.P., Lee M.H., Kim J.I., Pae S.-B., Kim M., Oh K., Cho K.-S., Kim S. (2020).

[bib3] Diouf M., Boureima S., Tahir D., Çağirgan M.I. (2010). Gamma rays-induced mutant spectrum and frequency in sesame. Turk. J. Field Crops.

[bib4] Dossa K., Diouf D., Cissé N. (2016). Genome-wide investigation of Hsf genes in sesame reveals their segmental duplication expansion and their active role in drought stress response. Front. Plant Sci..

[bib5] Dossa K., Wei X., Li D., Fonceka D., Zhang Y., Wang L., Yu J., Boshou L., Diouf D., Cissé N. (2016). Insight into the AP2/ERF transcription factor superfamily in sesame and expression profiling of DREB subfamily under drought stress. BMC Plant Biol..

[bib6] Dossa K., Diouf D., Wang L., Wei X., Zhang Y., Niang M., Fonceka D., Yu J., Mmadi M.A., Yehouessi L.W. (2017). The emerging oilseed crop Sesamum indicum enters the “Omics” era. Front. Plant Sci..

[bib7] Dossa K., Li D., Wang L., Zheng X., Liu A., Yu J., Wei X., Zhou R., Fonceka D., Diouf D. (2017). Transcriptomic, biochemical and physio-anatomical investigations shed more light on responses to drought stress in two contrasting sesame genotypes. Sci. Rep..

[bib8] Dossa K., Li D., Wang L., Zheng X., Yu J., Wei X., Fonceka D., Diouf D., Liao B., Cisse N. (2017). Dynamic transcriptome landscape of sesame (Sesamum indicum L.) under progressive drought and after rewatering. Genom. Data.

[bib9] Dossa K., Mmadi M.A., Zhou R., Zhang T., Su R., Zhang Y., Wang L., You J., Zhang X. (2019). Depicting the core transcriptome modulating multiple abiotic stresses responses in sesame (Sesamum indicum L.). Int. J. Mol. Sci..

[bib10] Dossa K., You J., Wang L., Zhang Y., Li D., Zhou R., Yu J., Wei X., Zhu X., Jiang S. (2019). Transcriptomic profiling of sesame during waterlogging and recovery. Sci. Data.

[bib11] Dossa K., Mmadi M.A., Zhou R., Liu A., Yang Y., Diouf D., You J., Zhang X. (2020). Ectopic expression of the sesame MYB transcription factor SiMYB305 promotes root growth and modulates ABA-mediated tolerance to drought and salt stresses in Arabidopsis. AoB Plants.

[bib12] Dubey S., Bist R., Misra S. (2017). Sodium azide induced mutagenesis in wheat plant. World J. Pharm. Pharmaceut. Sci..

[bib13] Duncan D.B. (1955). Multiple range and multiple F tests. Biometrics.

[bib14] Heslot H. (1977). Manual on Mutation Breeding: Technical Reports.

[bib15] IPGRI N. (2004).

[bib16] Jayabalan M. (1995).

[bib17] Li D.-h., Wang L.-h., Zhang Y.-x., Lv H.-x., Qi X.-q., Wei W.-l., Zhang X.-r. (2012). Pathogenic variation and molecular characterization of Fusarium species isolated from wilted sesame in China. Afr. J. Microbiol. Res..

[bib18] Li D., Liu P., Yu J., Wang L., Dossa K., Zhang Y., Zhou R., Wei X., Zhang X. (2017). Genome-wide analysis of WRKY gene family in the sesame genome and identification of the WRKY genes involved in responses to abiotic stresses. BMC Plant Biol..

[bib19] Mmadi M.A., Dossa K., Wang L., Zhou R., Wang Y., Cisse N., Sy M.O., Zhang X. (2017). Functional characterization of the versatile MYB gene family uncovered their important roles in plant development and responses to drought and waterlogging in sesame. Genes.

[bib20] Najeeb U., Mirza M., Jilani G., Mubashir A., Zhou W. (2012).

[bib21] Olawuyi O.J., Okoli S.O. (2017). Genetic variability on tolerance of maize (Zea mays L.) genotypes induced with sodium azide mutagen. Mol. Plant Breed..

[bib22] Payne R., Baird D., Cherry M., Gilmour A., Harding S., Lane P., Morgan G., Murray D., Soutar D., Thompson R. (2002). GenStat release 6.1 reference manual. Part 1. Summary. VSN Int..

[bib23] Smith R., Grando M., Li Y., Seib J., Shatters R. (2002). Transformation of bahiagrass (Paspalum notatum Flugge). Plant Cell Rep..

[bib24] Stewart D., Costa C., Dwyer L., Smith D., Hamilton R., Ma B. (2003). Canopy structure, light interception, and photosynthesis in maize. Agron. J..

[bib25] Student F.M.S. (2008). Evaluation of seed yield-related characters in sesame (Sesamum indicum L.) using factor and path analysis. Pakistan J. Biol. Sci..

[bib26] Tripathy S.K., Kar J., Sahu D. (2019). Advances in Plant Breeding Strategies: Industrial and Food Crops.

[bib27] Wang L., Li D., Zhang Y., Gao Y., Yu J., Wei X., Zhang X. (2016). Tolerant and susceptible sesame genotypes reveal waterlogging stress response patterns. PLoS One.

[bib28] Wang L., Zhang Y., Zhu X., Zhu X., Li D., Zhang X., Gao Y., Xiao G., Wei X. (2017). Development of an SSR-based genetic map in sesame and identification of quantitative trait loci associated with charcoal rot resistance. Sci. Rep..

[bib29] Wang D., Zhang L., Huang X., Wang X., Yang R., Mao J., Wang X., Wang X., Zhang Q., Li P. (2018). Identification of nutritional components in black sesame determined by widely targeted metabolomics and traditional Chinese medicines. Molecules.

[bib30] Wang Y., Zhang Y., Zhou R., Dossa K., Yu J., Li D., Liu A., Mmadi M.A., Zhang X., You J. (2018). Identification and characterization of the bZIP transcription factor family and its expression in response to abiotic stresses in sesame. PLoS One.

[bib31] Wei L., Zhang H., Duan Y., Li C., Chang S., Miao H. (2016). Transcriptome comparison of resistant and susceptible sesame (Sesamum indicum L.) varieties inoculated with Fusarium oxysporum f. sp. sesami. Plant Breed..

[bib32] Wei M., Liu A., Zhang Y., Zhou Y., Li D., Dossa K., Zhou R., Zhang X., You J. (2019). Genome-wide characterization and expression analysis of the HD-Zip gene family in response to drought and salinity stresses in sesame. BMC Genom..

[bib33] Weldemichael M.Y., Bayratsion Y.T., Sbhatu D.B., Abraha G.G., Juhar H.M., Birhan Kassa A., Sibhatu F.B., Berhe B.D., Sahle T.E., Mossa M.M. (2020). In vitro shoot regeneration of oil seed crop Sesamum indicum L. From seedling cotyledon explant to lay ground for genetic transformation in Ethiopia. Int. J. Agron..

[bib34] Weldemichael M.Y., Baryatsion Y.T., Sbhatu D.B., Gebresamuel Abraha G., Juhar H.M., Kassa A.B., Baraki Sibhatu F., Gebremedhn H.M., Gebrelibanos T.S., Mebrahtu Mossa M. (2021). Effect of sodium azide on quantitative and qualitative stem traits in the M2 generation of Ethiopian sesame (Sesamum indicum L.) genotypes. Sci. World J..

[bib35] Wu K., Liu H., Yang M., Tao Y., Ma H., Wu W., Zuo Y., Zhao Y. (2014). High-density genetic map construction and QTLs analysis of grain yield-related traits in Sesame (Sesamum indicum L.) based on RAD-Seq techonology. BMC Plant Biol..

[bib36] Yan W., Ni Y., Liu X., Zhao H., Chen Y., Jia M., Liu M., Liu H., Tian B. (2021). The mechanism of sesame resistance against Macrophomina phaseolina was revealed via a comparison of transcriptomes of resistant and susceptible sesame genotypes. BMC Plant Biol..

[bib37] You J., Zhang Y., Liu A., Li D., Wang X., Dossa K., Zhou R., Yu J., Zhang Y., Wang L. (2019). Transcriptomic and metabolomic profiling of drought-tolerant and susceptible sesame genotypes in response to drought stress. BMC Plant Biol..

[bib38] Zhang Y., Li D., Wang Y., Zhou R., Wang L., Zhang Y., Yu J., Gong H., You J., Zhang X. (2018). Genome-wide identification and comprehensive analysis of the NAC transcription factor family in Sesamum indicum. PLoS One.

[bib39] Zhang Y., Li D., Zhou R., Wang X., Dossa K., Wang L., Zhang Y., Yu J., Gong H., Zhang X. (2019). Transcriptome and metabolome analyses of two contrasting sesame genotypes reveal the crucial biological pathways involved in rapid adaptive response to salt stress. BMC Plant Biol..

[bib40] Zhou R., Liu P., Li D., Zhang X., Wei X. (2018). Photoperiod response-related gene SiCOL1 contributes to flowering in sesame. BMC Plant Biol..

